# Wormian Bone of the Orbit: A Case Report

**DOI:** 10.7759/cureus.3117

**Published:** 2018-08-07

**Authors:** Asad Rizvi, Joe Iwanaga, Rod J Oskouian, Marios Loukas, R. Shane Tubbs

**Affiliations:** 1 Medicine, St. Georges University School of Medicine, St. Georges, GRD; 2 Medical Education and Simulation, Seattle Science Foundation, Seattle, USA; 3 Neurosurgery, Swedish Neuroscience Institute, Seattle, USA; 4 Anatomical Sciences, St. George's University, St. George's, GRD; 5 Neurosurgery, Seattle Science Foundation, Seattle, USA

**Keywords:** wormian bones, supernumerary bones, sutural bones, orbit, imaging, orbital fractures, cranial sutures, fontanelles

## Abstract

Wormian bones are formed due to abnormal ossification centers in various locations in the skull. Genetic and/or environmental factors have been proposed to explain their formation. These bones can be normal anatomical variants or associated with a number of pathological conditions. The literature reports the most common locations of these bones as the cranial sutures, and reports of the presence of these bones in the orbit are rare. Clinically, these bones in the orbit can simulate fractures on imaging or can dislodge during surgery causing injury to the surrounding structures. Herein, we report a case of wormian bones of the orbit and discuss other reports from the literature.

## Introduction

Wormian, supernumerary, sutural, or intra-sutural bones are small isolated bones in various locations in the skull that are the result of abnormal ossification centers [1]. According to Romero-Reverón and Arráez-Aybar [2], these bones are named after the Danish anatomist and physician, Olaus Wormius, who first described them in 1643. Wormian bones can be present as normal anatomical variants or in association with a number of pathological conditions, e.g., osteogenesis imperfecta where these bones are part of the diagnostic criteria, craniosynostosis, hydrocephalus, cleidocranial dysostosis, cretinism, rickets, hypophosphatasia, and more [1, 3].

The exact cause of the formation of these bones is not clear and genetic and/or environmental factors have been implicated [1]. Hypotheses in favor of environmental factors include the observation that the Chinese population has the highest frequency of wormian bones, particularly in the posterior skull. It has been postulated that this could be due to more extended supine infant sleeping positions leading to constant pressure on the occipital region; this constant pressure might put a strain on the dura mater and expands the sutures [4, 5]. Others have noted a higher prevalence of wormian bones in intentionally deformed crania favoring an environmental cause for the formation of some of these bones [6].

Wormian bones are commonly reported in the cranial sutures, especially over the calvaria [1, 3, 5, 7]. The lambdoid and the coronal sutures are the most common locations among the sutures and the posterior fontanelle is the most common location for the fontanelles [1, 7]. While there are many reports of wormian bones in the cranial sutures and fontanelles, reports of these bones in the orbit are rare [8]. Knowledge of the presence of these bones in the orbit is vital as surgical manipulation can dislodge these bones causing injury to the surrounding structures [9]. Herein, we present a case of a wormian bone in the orbit and discuss other reports in the literature in an attempt to highlight their existence and clinical significance.

## Case presentation

**Figure 1:**
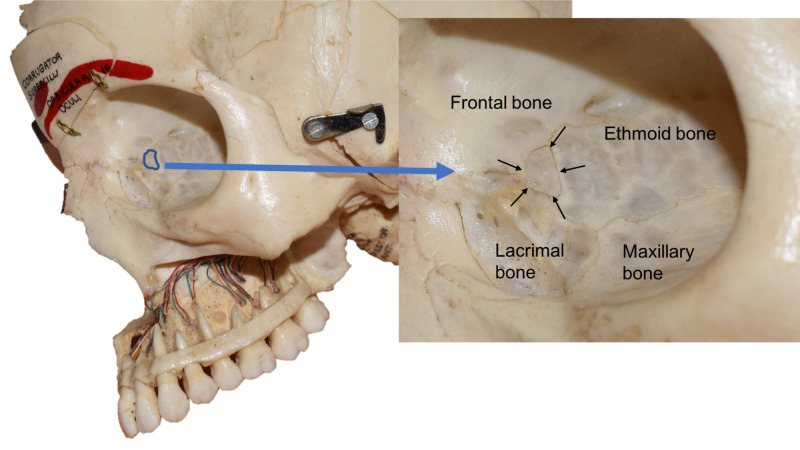
Left orbit from the skull specimen described in the present case report. The wormian bone is outlined on the left in blue. The right zoomed in view also shows this bone (arrows). Note the relationship of other regional bones such as the ethmoid and lacrimal bones.

The left orbit from an adult female skull was evaluated and found to have a small (4 x 5.5 mm) wormian bone (Figure 1). The skull was most likely Asian in origin. This very thin bone was roughly rectangular in shape and wedged between the lamina papyracea part of the ethmoid bone posteriorly, the frontal bone superiorly, and the lacrimal bone anteriorly. The skull from this specimen also had absence of the sagittal suture, a deviated septum, and an aerated left middle nasal concha. No other obvious anatomical variations of the skull were observed.

## Discussion

A recent study demonstrated that 53% (320/605) of pediatric patients had at least one wormian bone, mostly in the lambdoid suture [10]. Another study concluded that 8-15% of the population have at least one wormian bone [5]. However, reports of wormian bones in the orbit are rare.

Lateral and medial walls of the orbit

Frequencies of wormian bones in the lateral wall of the orbit have been reported as 12.5% [9]. In 2.5% of the samples, these bones were present on the right orbit in the frontozygomatic suture. In 5% of the samples they were observed in the sphenozygomatic suture (bilaterally in 3.75%), and 3.75% of the samples had a wormian bone in the sphenofrontozygomatic suture [9]. A study using a smaller sample reported wormian bones in 11.04% of the skulls examined. Other reports have also observed accessory ossicles in the sphenozygomatic sutures [8, 11, 12]. On the medial wall of the orbit, wormian bones have been reported in the frontoethmoidal suture [9, 13].

Roof and floor of the orbit

Wormian bones were observed on the roof of the orbit in the orbital plate of the frontal bone in 2.5% of the samples and at the junction of the lesser wing of the sphenoid and the orbital plate of the frontal bone in 1.25% of the samples [9]. In another study, 4.29% of the samples had wormian bones in the roof of the orbit located anterior to the lesser wing of the sphenoid [8]. These bones have been reported with a frequency of 2.5% on the floor of the orbit in the ethmoidomaxillary suture [9].

Finally, wormian bones have been found with a higher frequency in the orbit on right sides; it has been postulated that this could be due to the richer blood supply on the right [9].

An understanding of the anatomical variations in the orbit is clinically important as, for example, the wormian bones might simulate fractures on imaging of the orbit or may get dislodged during surgical manipulation causing injury to the nerves, blood vessels, and the eyeball [9, 14-16].

## Conclusions

There are reports of the presence of wormian bones in the cranial sutures and fontanelles in the literature. However, reports of these bones in the orbit are scarce. Here, we present a case of a wormian bone in the orbit and discuss other reports from the literature concerning the frequency and location of these bones in the walls, roof, and the floor of the orbit. Clinicians should be aware of the possible locations of these bones in the orbit when interpreting imaging and during surgery to avoid possible complications. Future studies can focus on the frequency and location of wormian bones in the orbit in different populations to further understand their distribution and perhaps etiology.
